# Structural properties of starch-chitosan-gelatin foams and the impact of gelatin on MC3T3 mouse osteoblast cell viability

**DOI:** 10.1186/s13036-017-0086-z

**Published:** 2017-11-21

**Authors:** Gregory E. Risser, Brittany L. Banik, Justin L. Brown, Jeffrey M. Catchmark

**Affiliations:** 1 Department of Engineering Science and Mechanics, The Pennsylvania State University, University Park, PA 16802 USA; 2 Department of Biomedical Engineering, The Pennsylvania State University, University Park, PA 16802 USA; 3 Department of Agricultural and Biological Engineering, The Pennsylvania State University, 109 Agricultural Engineering Building, University Park, PA 16802 USA

**Keywords:** Biofoam, Scaffold, Starch, Chitosan, Gelatin, Osteoblast cells

## Abstract

**Background:**

This study examines the effects of adding gelatin to a starch-chitosan composite foam, focusing on the altered structural and biological properties. The compressive modulus of foams containing different gelatin concentrations was tested in dry, wet, and lyophilized states. MC3T3 mouse osteoblast cells were used to test the composite’s ability to support cell growth. The stability of the foams in α-MEM culture media with and without cells was also examined.

**Results:**

It was found that for dry foams, the compressive modulus increased with increasing gelatin content. For foams tested in wet and lyophilized states, the compressive modulus peaked at a gelatin concentration of 2.5% and 5%, respectively. The growth of MC3T3 mouse osteoblast cells was tested on the foams with different gelatin concentrations. The addition of gelatin had a positive effect on the cell growth and proliferation.

**Conclusion:**

The composite foam containing gelatin improved cell growth and is only dissolved by the growing cells at a rate influenced by the initial concentration of gelatin added to the foam.

## Background

Biomaterials are useful for aiding the human body’s healing process [1]. In particular, natural biomaterials are gaining more interest due to their strong biocompatibility and ability to promote cellular adhesion [2]. Chitosan is an example of such a biomaterial. This is in large part due to its ability to form polyelectrolyte complexes with negative polyanions in solution at a low pH [3]. Chitosan is a product derived from chitin, a naturally occurring polysaccharide. To obtain chitosan from chitin, the chitin must be partially deacetylated. Deacetylated chitosan contains a D-glucosamine repeat unit, allowing chitosan to become cationic under acidic conditions when the amine is protonated [4]. Chitosan-based scaffolding has been studied with many other types of polymers. Some examples of these include silk fibroin, collagen, alginate, and gelatin [5–8].

A previous study found success in combining starch and chitosan to create a foam, where the starch acted as the polyanionic complex [9]. In this current study, gelatin was added to the composite so that its impact on foam mechanical behavior and cell growth dynamics could be examined. Gelatin is another well-studied material for tissue scaffolds because it is derived from collagen. Gelatin has free carboxyl groups that allow for bonding with the chitosan [8]. Gelatin-chitosan scaffolds have been synthesized and tested for various types of tissues, such as skin, bone and cartilage [10]. The mechanical properties resulting from combining gelatin and chitosan have also been studied, and it was found that increasing gelatin concentration improved the modulus of the chitosan gelatin blend [10]. However, results concerning the change in cell viability on the scaffolds are lacking [10].

This study combines potato starch, chitosan and gelatin to form a natural foam biomaterial. The structure, compression modulus, solubility and cell compatibility were studied to evaluate the applicability of the foam as a cell growth scaffold material. The gelatin and chitosan are desirable for their cellular adhesion and migration aiding properties as well as their ability to form polyelectrolyte complexes [8].

## Methods

### Foam preparation

The foam was prepared using a blend of potato starch (PS) (Sigma Aldrich 03967), chitosan (CS) (Sigma Aldrich 448,869, exhibiting a degree of deacetylation greater than 75.0%) and gelatin from porcine skin (Sigma Aldrich G1890). The CS solution was composed of water, 3.34% chitosan, and 1% *v*/v formic acid (Alfa Aesar 36,504, 88%). The CS solution was made by mixing 191 ml of water and 6.68 g of chitosan for 15 min at 80 °C. The temperature on the hot plate was then reduced to 45 °C and 2 ml of formic acid was slowly added to the solution. After mixing for 20 min at 45 °C, the solution was removed from the hot plate and mixing continued for another hour to ensure homogeneity. Once the final solution was made, it was capped to minimize evaporation of water.

Four distinct foams were made, each with a different concentration of gelatin. The compositions of each foam is shown in Table 1.

**Table 1:** Composition of foams

Name	Potato Starch (g)	3.34% Chitosan Solution (g)	Gelatin (g)	Microwave Time (s)
0.0% Gelatin Foam	2.5	2.5	0	47
2.5% Gelatin Foam	2.5	2.5	.125	52
5.0% Gelatin Foam	2.5	2.5	.25	60
10.0% Gelatin Foam	2.5	2.5	.5	60

Following the mixture of 2.5 g of PS and the prescribed gelatin amount in a polystyrene cup, 2.5 g of CS solution was added. These components were blended together until the final form was a homogenous paste. The blend was heated in a microwave for the amount of time indicated in Table 1. As the mixture was heated, the water contained in it transitioned to a vapor and caused the mixture to expand. A dry foam was obtained after the microwave treatment was completed. Longer microwave heating times were required when increasing amounts of gelatin were added to the foam. Without longer treatment times, the foams with higher amounts of gelatin did not solidify and instead were gel-like. This likely occurred as a result of the gelatin holding more water in the foam composition during heating.

The dry foams were tested as produced from the microwave. The wet foams were soaked for 24 h in nanopure DI water. The lyophilized foams were soaked for 24 h in nanopure DI water, frozen for 24 h, and then lyophilized.

### Pore size measurements

An optical microscope (Zeiss Axio A1m) with a 10× objective lens was used to measure the foam pore size distribution. Multiple images were captured using an image processing software (Axiovision) and a measure function was applied to the images to obtain pore size. The pore size measurements were then averaged.

### Compression testing

The foams were cut into rectangular prisms and then compressed using an Instron mechanical analyzer. During compression, a linear stress vs. strain behavior was initially observed. Once the curve began to exhibit exponential behavior, the test was stopped. At this point the material was crushed. The results were recorded using Bluehill software. The foams were tested under three different states: dry, wet, and lyophilized.

### Sterilization

Each of the foam scaffolds was sterilized via dry-autoclaving. The foams couldn’t be sterilized by an ethanol treatment because ethanol dissolved the foams. The foams were not steam-autoclaved to prevent water absorption and expansion. This was critical so that the foams were dry before placement into cell culture media, allowing them to absorb cell-containing media and facilitate cell attachment. The samples were placed into self-sealing sterilization pouches and were heated at 165 °C for an hour at 30 psi.

### Cell culture

MC3T3-E1 Subclone 4 mouse osteoblast cells were seeded onto the scaffolds. They were first removed from a nitrogen cooled freezer then thawed. The cells were cultured in α-MEM culture media (Thermo Scientific 41,061,029) supplemented with 10% fetal bovine serum and 1% penstrep. The cells were grown to 80% confluency before they were detached from the culture dish for seeding.

### Seeding cells

The dry foams were cut into scaffolds with dimensions of 1 cm length × 1 cm width × 0.5 cm height. Cells were seeded onto a scaffold by placing 2 ml of a cell-containing media mixture on the scaffold. The culture media was fully absorbed by the foams within 4 h. An additional 2 ml of supplemented α-MEM media was added to each well to completely cover the scaffold. The seeded scaffolds were then incubated.

For the MTT assay, samples were again cut into 1 cm × 1 cm × 0.5 cm pieces and seeded. The cells were suspended in 300 μl of media, creating a more concentrated cell solution to add to the foams. The foams were incubated for 4 h to ensure cell attachment. Media was added to cover the foams, and samples were incubated for 24 and 64 h.

### Immunofluorescence

A paraformaldehyde fixation buffer (FB) and permeabilization buffer (PB) were prepared for immunofluorescence studies. The FB was made with 3.7% paraformaldehyde in 1× phosphate buffered saline (PBS). The PB buffer was made with 1× PBS, 3% bovine serum albumin, and 0.1% Triton X-100. Excess media was removed with washes of 1× PBS to prevent issues during fluorescence imaging. The samples were fixed for 15 min and blocked with PB for 45 min. Samples were incubated for 30 min in the dark with Phalloidin 565 (1:1000) and DAPI (1:5000) to fluorescently stain actin and double-stranded DNA in the nuclei, respectively. Then the scaffolds were imaged using a Leica DM5500 upright microscope (Leica Microsystems, Buffalo Groove, IL) and analyzed with Leica LASX software.

### MTT assay

MC3T3 cells were seeded onto 1 cm × 1 cm × 0.5 cm foams of 0%, 2.5%, 5%, and 10% gelatin concentrations. Cells were incubated for 60 h at 37 °C. The scaffolds were moved to a new plate to make sure only the cells seeded onto the scaffold were assayed. 1.9 ml of culture media was added to each well along with 0.19 ml of MTT stock solution (3 ml sterile PBS and 3 mg MTT powder). Following a 4 h incubation, 1.9 ml of SDS solution were added to each well and the samples were incubated for an additional 4 h. A microplate reader was used to read the absorbance at 570 nm.

### Foam dissolution

Foams of the 4 different gelatin concentrations were cut into rectangular prisms for testing (*n* = 3). They were then submerged into either of 2 media: 1) α-MEM culture media (Thermo Scientific 41,061,029) supplemented with 10% fetal bovine serum and 1% penstrep and 2) α-MEM without FBS. The foams were incubated in the media for 7 days at 37 °C and dissolution was checked each day.

## Results

### Pore size measures

**Fig. 1:**
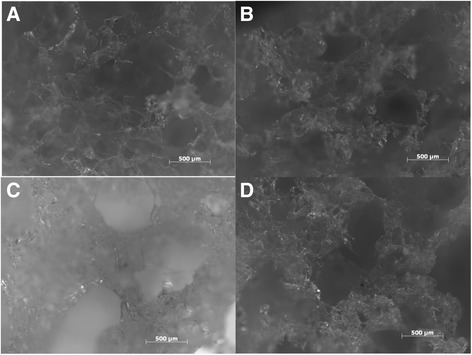
Composite foams containing (**a**) 0%, (**b**) 2.5%, (**c**) 5%, and (**d**) 10% gelatin. Images were taken using an optical microscope. **a** has pores of estimated pore sizes of 400 μm with thin walls. b has two pore sizes of 250 μm and 600 μm with walls thicker than that of the 0% gelatin. **c** and **d** have very large pores estimated to be 1500 μm along with pores in the walls measuring roughly 100 μm

Figure 1a-d depict microscope photographs of foams with different gelatin concentrations of 0%, 2.5%, 5%, and 10%, respectively. Table 2 summarizes approximate foam pore size. The control foam with no gelatin showed many pores, all approximately 400 μm in diameter. This foam had the largest free volume of the foams because of the large pores and thin walls. The 2.5% gelatin foam had pores around 250 μm in length as well as larger pores around 600 μm in length. Examination of the 5% gelatin foam revealed large pores with a diameter around 1500 μm. Looking closer however, smaller pores were seen in the thick walls of the foam. These smaller pores had a diameter of around 100 μm. The 10% gelatin foams exhibited a similar structure. Both the large and small pores in this foam were smaller than those exhibited by the 5% foam.

**Table 2:** Pore sizes of different foams

Name	Estimated Pore Length (microns)
0.0% Gelatin Foam	400
2.5% Gelatin Foam	250 and 600
5.0% Gelatin Foam	100 (within pore wall) and 1500 (large pores)
10.0% Gelatin Foam	100 (within pore walls) and 1500 (large pores)

### Compression tests

**Fig. 2:**
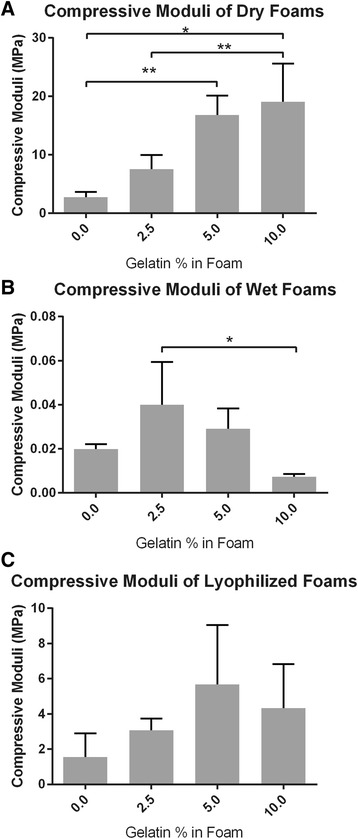
Compressive moduli of the foams with varying levels of concentration and in three different states. **a** shows the moduli of the foams in a dry state. **b** shows the moduli of the foams in a wet state. **c** shows the moduli of foams in a lyophilized state. Sample size of *n* = 3 and bars with * or ** indicates statistical significance of *p* < 0.05 or *p* < 0.01, respectively between the two foams. Significance was obtained using ANOVA and Tukey post hoc analysis

The compression strength of the foams with varying levels of gelatin were tested under different conditions. The four different compositions had the following concentrations of gelatin: 0%, 2.5%, 5%, and 10%. Using the stress vs. strain data, compressive moduli were calculated and shown in Fig. 2.

As seen in Fig. 2a-c, the values of the dry compression modulus increase as the gelatin concentration increases. There is a significant increase in modulus with the addition of 2.5% and 5% gelatin but no significant change for 10% gelatin concentration. As expected, the dry foams exhibited a much higher compression modulus than the wet foams. Compression modulus data from the wet foams exhibited a large variation in measured values, making comparison difficult. The addition of 10% gelatin, however, clearly reduced the wet modulus. Lyophilized modulus values showed a general increase with gelatin concentration but all values are greater than the wet modulus values and comparable to the control (no gelatin added) dry modulus.

**Fig. 3:**
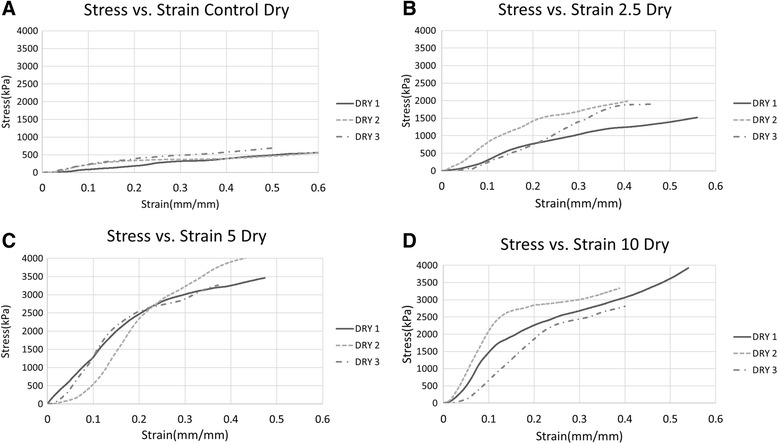
Graphs of the compression data for the foams in a dry state with (**a**) 0%, (**b**) 2.5%, (**c**) 5%, and (**d**) 10% gelatin. Generally, as the concentration of the gelatin increased, the modulus also increased

Figure 3 depicts stress vs. strain diagrams under compressive loading for dry foams produced using different gelatin concentrations. Virtually all plots show an initial low slope where little force (less than ~50 kPa) was required to produce a strain (~0.03–0.05). In this strain region, the entire cross sectional area of the foam was not being compressed due to shape irregularity. After a certain strain was reached, the entire cross sectional area began to resist the compression and the slope of the stress-strain curve increased and became linear. The extent of the linear region varied across the sample composition types.

Stress vs. strain curves were not smooth and instead, wavered as the test progressed. This was likely caused by the architecture of the foams and how it affects the compressing process. Due to the natural structure of the foams, there is wide variation on pore sizes and distributions. As the foam is compressed, the weakest region of the foam may yield allowing the applied stress to be redistributed at which point the resistance to compression increases.

The linear portion of the stress vs. strain plot was used to calculate the compression modulus of each foam. When performing this calculation, an effort was made to use the same strain range to enable more relevant comparison of the behavior of the foam samples. The compressive properties of the PS/CS/G foams have a greater modulus than other foams made of just CS and gelatin separately [11]. It should be noted that the wet samples exhibited resiliency thus the calculated modulus values represent a true modulus. Dry samples, however, began to crush at low strain values. Thus the term ‘modulus’ in this case may be less accurate, as the behavior is not reversible [12].

### Images of cell growth

**Fig. 4:**
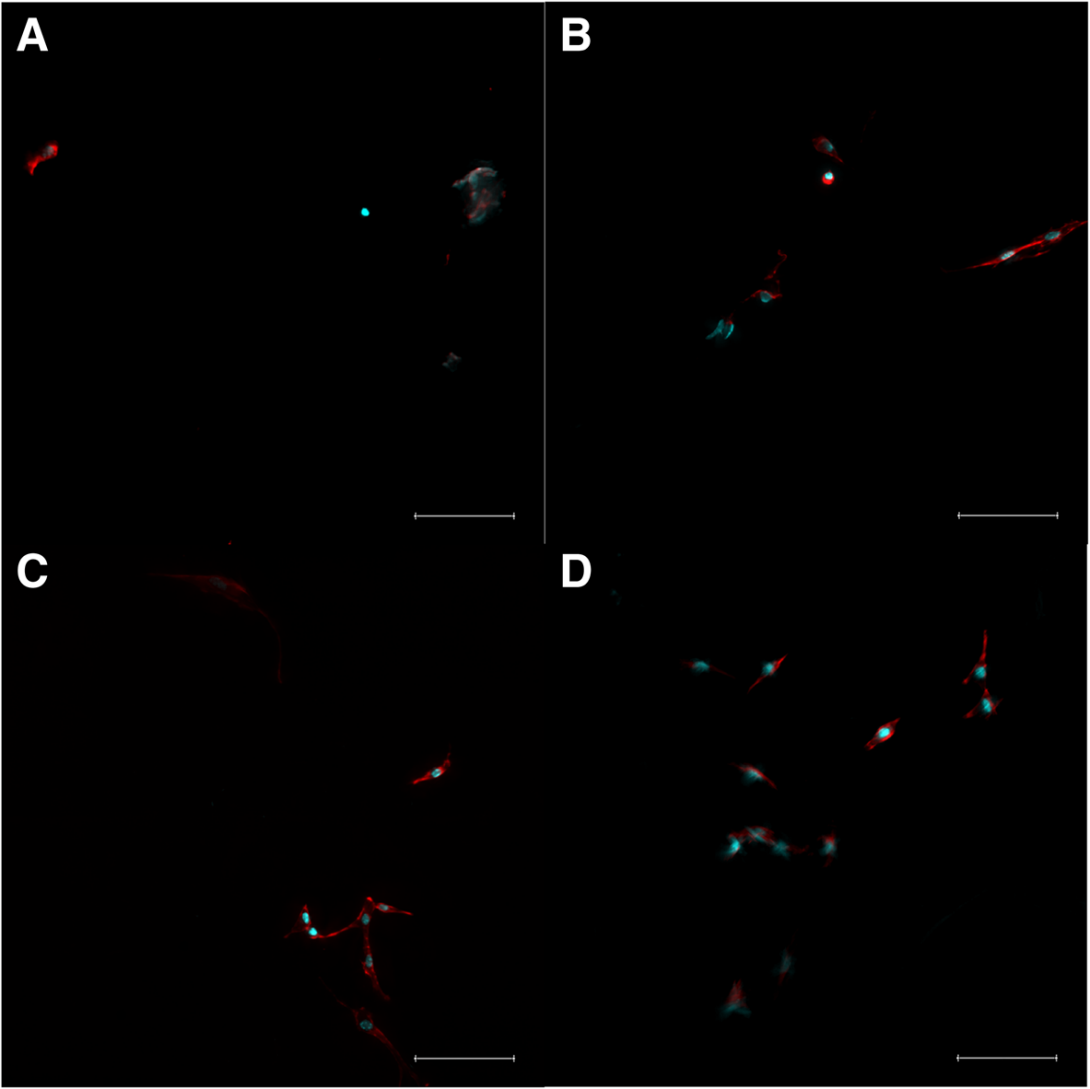
Cells were stained with Phalloidin and DAPI to illustrate cell actin (red) and nuclei (blue), respectively. Fluorescence images of cells grown on foams with different gelatin concentrations are shown: (**a**) 0%, (**b**) 2.5%, (**c**) 5%, and (**d**) 10%. Scale bar is 100 μm

After cells were seeded onto the scaffolds, they were incubated for 24 h before imaging. Figure 4a-d depict cells which have been stained with Phalloidin and DAPI to highlight the actin in red and the nuclei in blue [13]. Figure 4a is the foam with 0% gelatin and shows a reduced number of cells. The shape in the upper right hand corner of the image is most likely an artifact. Figure 4b is the foam with 2.5% gelatin added, which shows a slightly higher number of cells. Figure 4c is the foam with 5% gelatin added, and Fig. 4d is with 10% gelatin added. Again, with the 5% and 10% gelatin concentrations, the trend of increasing cells continued. Most of the cells in the images are long and stretched. These results showed that the combination of all three components (CS, PS, and G) form a biocompatible material capable of supporting cellular growth. These images qualitatively indicate that a higher concentration of gelatin in the foams can be correlated to better cell viability. To quantify the data, an MTT assay was performed to measure the amount of cellular activity per foam scaffold.

**Fig. 5:**
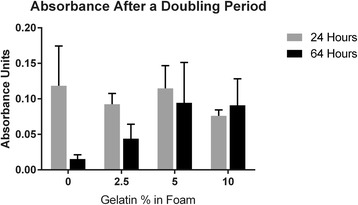
MTT assay results at 24 and 64 h of cell growth. The cells seeded onto the 10% gelatin foam were the only ones to have an increase in absorbance with an increase in time. The other groups showed a lesser amount at 64 h. Mean values were plotted and error bars represent the standard deviation. Sample size of n = 3 for 24 h group and *n* = 4 for 64 h group

Cellular activity was determined through an MTT assay conducted at 24 h and 64 h. MC3T3 cells have a doubling rate of 38 h, therefore after 64 h the cells had enough time to double and show a growth pattern, illustrated in Fig. 5. The values for the 24 h time point were closer to the same amount than compared to the values at the 64 h time point. The 0% gelatin and 5% gelatin foams had higher values at approximately 0.11 AU, while the 2.5% and 10% had slightly lower values of 0.09 AU and 0.07 AU. After 64 h, the 5% and 10% foams had values close to each other (approximately 0.09 AU) and were much higher than the 0% and 5% gelatin foams (0.015 AU and 0.04 AU).

### Foam dissolution

**Fig. 6:**
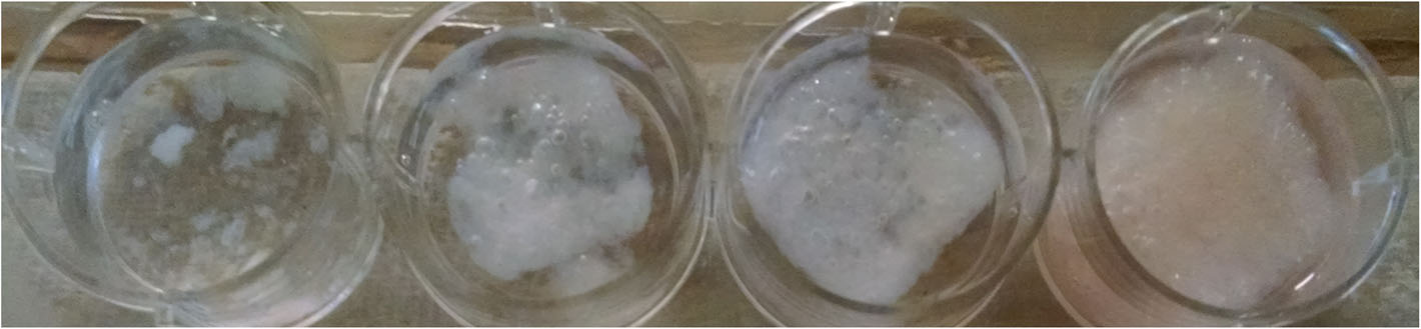
Image showing the differences of foam integrity after 64 h. From left to right, they are the 0% gelatin foam, 2.5% gelatin foam, 5% gelatin foam, and 10% gelatin foam

After cells had been seeded onto the scaffold, the scaffold started to dissolve. A noticeable trend occurred as the scaffolds dissolved. The scaffolds containing less gelatin degraded much more quickly than the scaffolds with higher gelatin composition as shown in Fig. 6. The 0% gelatin foam was severely degraded and only pieces of the foam remained. The 2.5% gelatin foam maintained its integrity better, but still showed moderate degradation with visible fractures. The 5% and 10% gelatin foams showed little to no degradation at all.

A dissolution experiment was set up to determine why the foams were breaking apart. Foams of the 4 different gelatin concentrations were placed into α-MEM containing FBS and α-MEM without FBS and incubated at 37 °C for 7 days. Each day the foams were examined for signs of dissolution. For each of the 7 days, the foams of all gelatin concentrations showed no visible degradation in either sets of α-MEM. This suggests that the cells attached to the foam were responsible for its degradation.

## Discussion

### Compression modulus

As the gelatin concentration increased, the compression modulus generally increased. This holds true for the dry foams but not for the wet and lyophilized foams. The increase in modulus can be explained by three factors. First, the addition of gelatin has been shown to improve the mechanical properties of starch-gelatin films analyzed under an applied tensile stress by Tongdeesoontorn et al. [14]. This may arise from the mechanical properties of gelatin itself as well as the interactions between gelatin and the starch. The amount of improvement observed by Tongdeesoontorn et al. decreased with increasing humidity. In the study, the starch did not contain anionic groups and improvement was believed to be associated with interactions between the OH^−^ and NH^3+^ groups. Such interactions may be sensitive to the presence of water that has a high affinity to the OH^−^ groups. Second, gelatin is known to undergo a dramatic change in its mechanical properties as the hydration level increases above ~25% as the gelatin transitions from the glassy to the rubbery state [15]. Thus, under fully hydrated conditions, the positive contribution of the gelatin itself to the mechanical properties of the foam may be substantially reduced. Third, as the gelatin concentration increased, the foams became denser. This result is similar to the results given by Zheng et al. [11]. They found that as the ratio between gelatin and chitosan increased, so did the compressive modulus in the dry foams. This is further corroborated by the images shown of the pore sizes (Fig. 1). The pore walls are thicker in the higher gelatin concentration foams than those of lower concentrations.

Having an increase in the compression modulus of the foams with an increase in gelatin concentration is quite beneficial. It provides a very simple method to increase the compression modulus of the foams. The compression modulus of wet foams decreased at the highest gelatin concentration of 10%. In addition to the reasons described above, this may also occur because the presence of increasing concentrations of positive gelatin begins to interfere with the electrostatic interactions between the more abundant positive amine groups on the chitosan and the negative phosphate groups on the starch. The chitosan used here exhibits a degree of deacetylation greater than 75%, indicating that at least 3 of 4 glucans in the polysaccharide contain an NH^3+^ group. This is a much higher polymer charge density than that exhibited by gelatin whose positive charge is associated with the presence of arginine, lysine and histidine amino acids that are present in the composition at a percentage of 8%, 4% and <1%, respectively [16]. These positive charges will cause gelatin to interact with the starch, precluding some amount of interaction with the chitosan reducing the overall degree of electrostatic complexation. In the hydrated state, electrostatic complexation may have a much larger influence on the mechanical properties of the composite since the presence of water gives the polymers mobility. Hydrogen bonding (that would be present in addition to electrostatic bonding in the dry state) is disrupted by water causing applied stresses to be resisted more by the polymers in complexation. Thus, composites containing higher amounts of gelatin and less complexation are expected to have a lower compression modulus when wet. This hypothesis, and the other observations above, may also explain the performance of the lyophilized foams.

### Cell viability

The results of the MTT assay shows that the number of live cells is positively affected by the proportion of gelatin added. One possible reason for this outcome is the change in shape the foams undergo as more gelatin is added. The control foams and 2.5% gelatin foams have uniform pores in the material and thin walls. The 5% and 10% gelatin composites have large pores with thick walls. The thick walls however are made of small pores around 100 μm. The combination of large and small pores may be beneficial to cell seeding and attachment. The large pores allow for the cells to move with the liquid, where they can attach well to the smaller pores of the foam. There are multiple types of acceptable pores sizes for different tissues; however for osteoblast cells there seems to be a time dependency that also plays a role [17]. One study found that when seeding bone cells onto a scaffold within 24–48 h, pore sizes around 120 μm encouraged the best growth. After 48 h, pores around 325 μm contained more cells [17]. Based on these results, it can be hypothesized that due to the variable pore sizes in the scaffold, cells might grow quicker in the smaller pore sizes initially. As the incubation period continues however, the larger pores allow for higher levels of cell migration. Another possible reason for the increase in cell viability would relate the material properties of gelatin. Gelatin has the RGD sequence Arg-Gly-Asp from collagen contained within it [18]. This sequence encourages cellular adhesion, resulting in composites with higher amounts of gelatin adhering more cells. This is reflected in the larger amount of cellular activity seen in the 5% and 10% gelatin foams.

### Foam dissolution

As the cells grew in the scaffolds, the scaffolds degraded. The rate of dissolution in the foams decreased with increasing gelatin content. To help determine the cause of the dissolution process, a dissolution test of the scaffolds was performed. In this test, scaffolds of varying gelatin concentrations were placed into α-MEM media that had FBS and α-MEM that didn’t have FBS. Both sets of foams showed very little degradation over a period of 7 days. Therefore, it is quite likely that the addition of MC3T3 cells caused the foams to break down. A possible cause of this could be explained by the production of amylase enzymes by the MC3T3 cells. If an amylase enzyme is being produced by the cells, it could be hydrolyzing the starch in the foams, causing them to dissolve. This result suggests that these foams may be bioabsorbable and the rate of bioabsorbability is dependent on the concentration of gelatin. The affinity of gelatin to starch may cause the gelatin to coat some of the starch making it inaccessible to the enzymes. Another factor may be the increased pore sidewall thickness. It may take longer for enzymes to penetrate the thicker foam pore sidewall structures reducing the rate of bioabsorption.

## Conclusions

The effects of adding gelatin to a PS and CS composite foam were tested and reported in this study. Adding gelatin to the mixture resulted in an increase in compressive modulus in the dry foam. The foams with 0% gelatin had a modulus of 2.72 MPa and increased to 19.06 MPa with a 10% gelatin concentration. Once wet and/or lyophilized, the upward trend of the compressive modulus peaked at 2.5% and 5% gelatin concentrations and then decreased. The addition of gelatin also resulted in better growth and activity of MC3T3 cells. The gelatin concentrations that had the best cellular growth were 5% and 10%. The amount of cells living after a 64 h period was higher in the 5% and 10% foams. Foams were also found to degrade when cells were growing but not in any of the buffers used in cultivation. This result suggests that the foams are bioabsorbable. Foams with increasing gelatin concentrations degraded more slowly, suggesting that control over foam bioabsorption is possible by varying gelatin concentration.
